# Hypothyroidism among patients with glioblastoma multiforme

**Published:** 2018-07-06

**Authors:** Morteza Faghih-Jouybari, Soheil Naderi, Sara Mashayekhi, Tahereh Padeganeh, Sina Abdollahzade

**Affiliations:** 1 Department of Neurosurgery, Shariati Hospital, Tehran University of Medical Sciences, Tehran, Iran; 2 Department of Neurosurgery, School of Medicine, Jiroft University of Medical Sciences, Jiroft, Iran; 3 Department of Maxillofacial Surgery, Shariati Hospital, Tehran University of Medical Sciences, Tehran, Iran; 4 Department of Neurosurgery, Rajaayi Hospital, Qazvin University of Medical Sciences, Qazvin, Iran

**Keywords:** Glioblastoma Multiforme, Hypothyroidism, Cranial Irradiation

## Abstract

**Background:** Patients with glioblastoma multiforme (GBM) are prone to various metabolic changes such as hypothyroidism. The present study was planned to assess the frequency of hypothyroidism in these patients.

**Methods:** Fifty-two patients with GBM were included. All of them had been treated by tumor resection followed by cranial irradiation. Thyroid function was assessed by measurement of serum thyroid stimulating hormone (TSH), free thyroxin (FT4), and free triiodothyronine (FT3).

**Results:** There were 33 men and 19 women. The average age was 52.4 ± 12.8 years. Among these, 32 (61%) had normal thyroid function test, whereas 4 (8%) had subclinical hypothyroidism, 5 (10%) had overt primary hypothyroidism, and 11 (21%) had secondary hypothyroidism. Sixteen patients (31%) needed thyroid hormone replacement therapy.

**Conclusion:** Hypothyroidism is relatively prevalent in patients with treated GBM. Regular thyroid function test is advised to aid the introduction of appropriate hormone replacement therapy.

## Introduction

Glioblastoma multiforme (GBM), the most common primary brain malignant tumor, frequently have a variety of neurological deficits. In addition, side effects of cranial irradiation, chemotherapy, antiepileptics, and surgical interventions make the patients more susceptible to metabolic derangements. Even subtle dysfunction of other organs could potentially cause deleterious effects on their general health.

Thyroid gland influences almost the whole metabolic processes in the body through its hormones. The thyroid hormones triiodothyronine (T3) and thyroxine (T4) facilitate some of the most fundamental physiologic processes in the body, including substrate use, energy expenditure, control of body temperature, growth, and development. Deficiency of thyroid hormones (hypothyroidism) can cause depression, fatigue, lethargy, ﬂuid retention, constipation, cold intolerance, and proximal muscle weakness. If untreated, it can lead to serious adverse health effects and ultimately death.^   1 ^

Usually, the clinical manifestations of hypothyroidism are vague, and can be disguised by sequels of surgery, radiotherapy, and chemotherapy in patients with GBM. Therefore, active pursuit of endocrine abnormalities is mandatory in this group of patients. 

As data concerning thyroid function in patients with GBM are scarce, our study aimed to define the frequency of hypothyroidism in these patients.

## Materials and Methods

The study pool consisted of consecutive adult (> 18 years) patients with established GBM defined by the pathological report, undergone surgery and radiotherapy. Cases were recruited from the outpatient neurosurgery clinic of Shariati University Hospital, a tertiary referral hospital in Tehran, Iran, from April 2014 to August 2016. 

Because of their confounding effects on thyroid function, we excluded patients on amiodarone or lithium, those with preexisting pituitary or thyroid disease, and any history of acute illness other than brain tumor less than 2 months from the day of recruitment. Consequently, 52 patients with GBM were included in the present study. Informed consent was obtained from all participants.

The duration between radiotherapy and the laboratory testing was at least 6 months. Thyroid stimulating hormone (TSH), free thyroxin (FT4), and free triiodothyronine (FT3) were measured using electrochemiluminescence immunoassay, and their normal references were deﬁned as 0.27-4.2 μIU/ml, 12.0-22.0 pmol/l, and 3.25-6.80 pmol/l, respectively. Subclinical hypothyroidism was deﬁned as serum TSH > 4.2 μIU/ml and normal FT4 level (12.0-22.0 pmol/l). Overt primary hypothyroidism was deﬁned as serum TSH > 4.2 μIU/ml and a low FT4 level (< 12.0 pmol/l). Secondary hypothyroidism was defined as TSH < 4.2 μIU/mL and a low FT4 level (< 12.0 pmol/l).

Continuous variables were expressed as mean ± standard deviations. Differences of variables were analyzed using t test. Statistical analyses were carried out via the SAS statistical analysis software package (version 9.1, SAS for Windows, SAS Institute, Cary, NC, USA).

## Results

**Figure 1:**
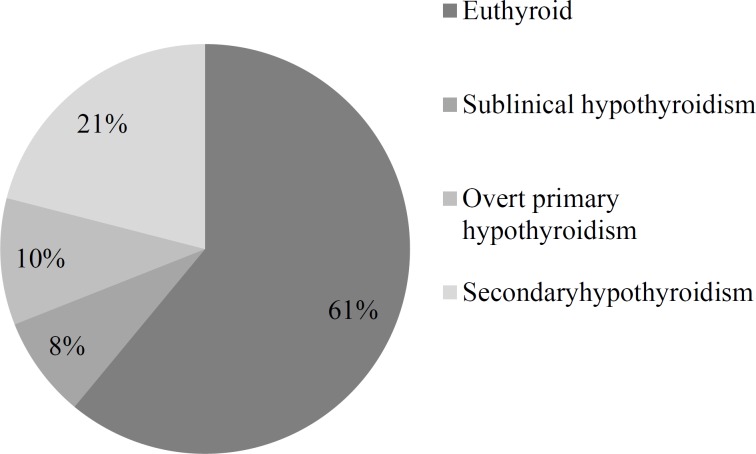
The frequency of thyroid dysfunction among patients with glioblastoma multiforme (GBM)

Fifty-two patients with GBM completed biochemical analysis for hypothyroidism. There were 33 men and 19 women. The age range was 23-78 years with the average of 52.4 ± 12.8. Among these, 32 (61%) had normal thyroid function test, whereas 4 (8%) had biochemical subclinical hypothyroidism (TSH > 4.2 mIU/l with normal FT4 levels), 5 (10%) had overt primary hypothyroidism, and 11(21%) had secondary hypothyroidism (TSH < 5.5 mIU/l with FT4 levels < 0.9 ng/dl). The prevalence of secondary and overt primary hypothyroidism together was 31% (Figure 1).

The mean age was 54.89 ± 8.85, 55.16 ± 10.28, 49.00 ± 13.12, and 51.32 ± 14.44 years in patients with normal thyroid function, secondary hypothyroidism, overt primary hypothyroidism, and subclinical hypothyroidism, respectively. There were no significant differences regarding age and gender among individuals with normal thyroid function and hypothyroidism.

## Discussion

In our study, 16 patients (31%) had overt hypothyroidism, and needed hormonal replacement therapy. Of these patients, 11 (21%) had secondary hypothyroidism and 5 (10%) had primary hypothyroidism. So, a considerable portion of patients had serious thyroid hormone deficiency. Our results were similar to survey by Merchant, et al. that 27% of patients with low-grade glioma needed thyroid hormone replacement after one year of treatment.^        2 ^

Secondary hypothyroidism might be caused by different mechanisms. Brain radiotherapy is a well-recognized cause of pituitary dysfunction. Radiation-induced anterior pituitary hormone deficiencies are common complications of brain tumor treatment.^        3 ^ They are irreversible and progressive and negatively impacts on quality of life. The pathophysiology of radiation-induced damage is not well understood. Chieng, et al. concluded that direct injury to the pituitary cells, rather than reduced blood flow, is the major cause of progressive pituitary dysfunction after fractionated cranial irradiation.^        4 ^ In a study by Kyriakakis, et al., of 107 adults with brain tumor and history of radiotherapy, 12% had secondary hypothyroidism. They advised that early detection and appropriate replacement of hormones is essential to improve quality of life.^        5 ^ A review by Appelman-Dijkstra, et al. showed that among 813 patients who underwent cranial irradiation, 25% developed TSH deficiency.^   6 ^

Surgical intervention itself might impair the tumor site as well as surrounding brain tissue and the pathway of access. Subsequently, pituitary dysfunction may ensue by mechanisms similar to brain trauma.^        7 ^ Schneider, et al. studied 68 adult patients with non-pituitary intracranial tumor, and found secondary hypothyroidism in 17.7% of them. Interestingly, hypopituitarism was more common in patients who underwent neurosurgery compared to those with radiotherapy or chemotherapy. They concluded that brain surgery had a major role in the development of hypopituitarism.^        8 ^

Moreover, other causes of hypothalamic-pituitary damage must be considered.^   9 ^ The tumor itself might be a source of damage by mass effects or inﬁltration. Additionally, metastatic destruction of the hypothalamus and/or pituitary gland have been described in some cases. 

Primary hypothyroidism was less common than secondary hypothyroidism in our study. Again, radiotherapy might be a probable cause. When treating malignant brain tumors with adjuvant irradiation, portions of the thyroid gland may receive scatter radiation doses, and potentially primary hypothyroidism may occur. Bhandare, et al. retrospectively reviewed the data from 197 patients treated with radiotherapy for head-and-neck tumors, and primary hypothyroidism was observed in 40 patients (20.3%).^        10 ^

## Conclusion

Hypothyroidism is relatively prevalent in patients with treated GBM. Regular testing is, thus, important to achieve timely diagnosis, and to aid the introduction of appropriate hormone replacement therapy to prevent or ameliorate consequences of hypothyroidism.

## Notes:

**How to cite this article:** Faghih-Jouybari M, Naderi S, Mashayekhi S, Padeganeh T, Abdollahzade S. Hypothyroidism among patients with glioblastoma multiforme. Iran J Neurol 2018; 17(3): 149-51.
